# Comparative Performance of Haptic Virtual Simulation vs. Conventional Training in Class V Cavity Preparation: A Paired In Vitro Study

**DOI:** 10.3390/dj14020109

**Published:** 2026-02-13

**Authors:** Aitor Basterra López, Sebastiana Arroyo Bote, Ángel Arturo López-González, Raúl Cuesta Román, Joan Obrador de Hevia, Pere Riutord-Sbert

**Affiliations:** School of Dentistry, ADEMA University School, 07009 Palma, Spain; a.basterra@eua.edu.es (A.B.L.); s.arroyo@eua.edu.es (S.A.B.); r.cuesta@eua.edu.es (R.C.R.); j.obrador@eua.edu.es (J.O.d.H.); p.riutord@eua.edu.es (P.R.-S.)

**Keywords:** haptic virtual simulation, virtual reality, dental education, preclinical competency, operative dentistry

## Abstract

**Background:** Haptic virtual simulation (HVS) has emerged as a promising tool in dental education, yet evidence comparing its performance to conventional preclinical training remains limited. Establishing its effectiveness is essential to support its integration into competency-based curricula. Objective: The aim of this study was to compare Class V cavity preparations performed using conventional training on extracted teeth with those performed using a haptic virtual simulator, evaluating preparation time and cavity volume. **Methods:** Sixty-one extracted human molars were digitized using cone-beam computed tomography (CBCT) to generate corresponding virtual replicas. A calibrated operator prepared 122 standardized Class V cavities (61 real and 61 virtual). The simulator automatically recorded preparation time and cavity volume. For natural teeth, cavity volume was calculated by digital superimposition of pre- and post-operative STL models using Blender. Paired means were compared using Student’s t-test (α = 0.05). **Results:** Preparation time was significantly shorter when using HVS compared with the conventional method (*p* < 0.001). Virtual preparations resulted in slightly larger cavity volumes than real preparations, with a statistically significant yet clinically small difference (*p* = 0.047). **Conclusions:** Haptic virtual simulation enables more time-efficient Class V cavity preparation while producing cavity volumes comparable to those obtained through conventional training. These findings support the implementation of haptic simulators as a valid and effective complement for preclinical skill acquisition in operative dentistry.

## 1. Introduction

The development of psychomotor skills in preclinical training is essential for dental students to achieve a safe and effective transition to clinical practice. Traditionally, operative dentistry curricula have relied on manikin-based simulation and resin typodont teeth to teach fundamental procedures such as cavity preparation and tooth tissue manipulation [1,2]. Although widely used, conventional simulation has important limitations: typodont materials do not accurately reproduce the hardness and tactile properties of enamel and dentin, they provide limited anatomical variability, and they demand intensive supervision from faculty to ensure accurate feedback and assessment [3,4].

Advances in digital technology have led to the integration of virtual simulation and haptic feedback systems into dental education. Haptic virtual reality simulators (HVRSs) enable three-dimensional visualization, force feedback, and instantaneous performance assessment, offering an interactive and standardized learning environment [5,6]. Several studies have demonstrated that HVRSs can reduce task completion time [7], improve surface smoothness and internal geometry of preparations [8], and enhance training consistency in operative dentistry exercises [9]. Systematic reviews further support their potential to complement or augment conventional simulation in developing fine motor skills in early-stage learners [10].

Despite these advances, key questions remain. Evidence comparing the equivalence between virtual and conventional training—particularly regarding quantitative outcomes such as cavity volume, cutting efficiency, and geometric accuracy—remains limited. For example, while some studies report improved performance after HVRS training [7,9], others show that immersive VR headsets used in conjunction with haptic simulators may hinder the accuracy of access cavity preparations due to perceptual overload [11]. Furthermore, few investigations have directly measured the amount of tooth structure removed using digital volumetric analysis, a metric essential for validating whether virtual preparations reflect realistic operative behavior. In addition, successful curriculum integration of HVRSs requires strong evidence that skills acquired in the virtual environment transfer reliably to physical tooth preparations.

Given these considerations, this study aims to compare Class V cavity preparations completed on extracted teeth and on a haptic virtual simulator by the same calibrated operator. By evaluating two key performance indicators—preparation time and cavity volume—this research seeks to contribute robust quantitative evidence regarding the utility of haptic simulation for competency development in operative dentistry.

## 2. Materials and Methods

### 2.1. Study Design

A paired in vitro comparative study was conducted to evaluate the performance of conventional preparation on extracted human teeth versus preparation using a haptic virtual simulation system. The study followed methodological standards commonly applied in preclinical dental education research [12,13,14].

#### 2.1.1. Sample Selection and Preparation

Sixty-one extracted human molars free of caries, restorations, cervical defects, or structural damage were obtained from a certified tissue bank. Teeth were cleaned of calculus using ultrasonic instrumentation and stored in a 0.1% thymol solution until use. Each tooth was embedded in photopolymerizable acrylic resin up to the cemento-enamel junction to simulate clinical positioning [15].

#### 2.1.2. Digitalization Procedure

All teeth were scanned using cone-beam computed tomography (CBCT) (MyRay Hyperion X9, Cefla, Imola, Italy). Scan parameters were standardized (FOV 5 × 5 cm; voxel size 75–100 μm). CBCT volumes were exported in DICOM format and converted to STL using InVesalius 3.1 (CTI, Campinas, Brazil). Thresholding parameters were adjusted manually to isolate dental hard tissues following recommended segmentation techniques [16].

Each STL file was imported into the simulator software (Virteasy Editor, HRV Simulation, Paris, France) to generate a corresponding virtual tooth model. At the time of data collection, the specific internal software version/build number was not recorded. The system corresponded to the commercially available Virteasy Dental V2 platform provided by HRV Simulation.

### 2.2. Cavity Preparation Procedures

#### 2.2.1. Conventional Preparation

A calibrated operator performed all Class V preparations on the 61 natural molars. The standardized cavity design followed classical operative criteria: kidney-shaped outline, 4.0 mm mesiodistal width, 1.5 mm occlusogingival height, 1.5 mm axial depth, and a 1 mm circumferential enamel bevel. Similar standardization approaches have been recommended in operative dentistry research [17].

Three diamond burs compliant with ISO 6360 standards were used sequentially (Komet^®^, Lemgo, Germany): round diamond bur (ISO 534–012, coarse grit), inverted cone bur (ISO 534–012, coarse grit), and needle bur (ISO 504–014, extra-fine grit) for bevels [ISO 6360-1]. Preparation time was recorded with a digital stopwatch [18].

Following preparation, teeth were rescanned using the same CBCT protocol to obtain postoperative STL files.

#### 2.2.2. Virtual Preparation

The same operator performed the Class V preparations on the 61 virtual tooth models using a haptic virtual reality simulator (Virteasy^®^, HRV Simulation, Paris, France). Bur shape, size, and sequence replicated those used in the real preparations. The simulator automatically recorded:

- preparation time (min)
- total removed volumetric mass (mm^3^)

Haptic force feedback was set to manufacturer-recommended calibration values to approximate the tactile sensations of enamel and dentin cutting.

### 2.3. Volumetric Analysis of Real Cavities

Cavity volume from natural teeth was quantified using 3D digital subtraction. Pre- and post-operative STL files were imported into Blender 3.1 (Blender Foundation, Amsterdam, The Netherlands). Models were aligned manually in three spatial planes, following established superimposition methodologies used in dental morphology research [19].

Boolean subtraction (Difference function) was applied to isolate the cavity volume, which was quantified using the “3D-Print Toolbox” volumetric function (mm^3^). Manual trimming of artefacts was performed when required.

To ensure the reliability and reproducibility of the volumetric measurements, a quality control procedure was implemented. A random subsample comprising 10% of all STL model pairs (pre- and post-operative scans) was re-analyzed by the same investigator after a two-week interval. Intra-operator reliability was assessed by repeating the alignment, segmentation verification, and Boolean subtraction procedures. The resulting intraclass correlation coefficient (ICC) exceeded 0.90 for repeated measurements, indicating excellent repeatability of the digital workflow. Additionally, all CBCT acquisitions were performed using identical imaging parameters, and simulator logs were automatically recorded to avoid transcription errors.

### 2.4. Outcome Variables

Two primary outcomes were assessed:

1. Preparation time (minutes):
  ○ manually recorded for conventional preparations
  ○ automatically logged for virtual preparations

2. Cavity volume (mm^3^):
  ○ digitally calculated for real cavities
  ○ automatically logged for virtual cavities

**Figure 1:**
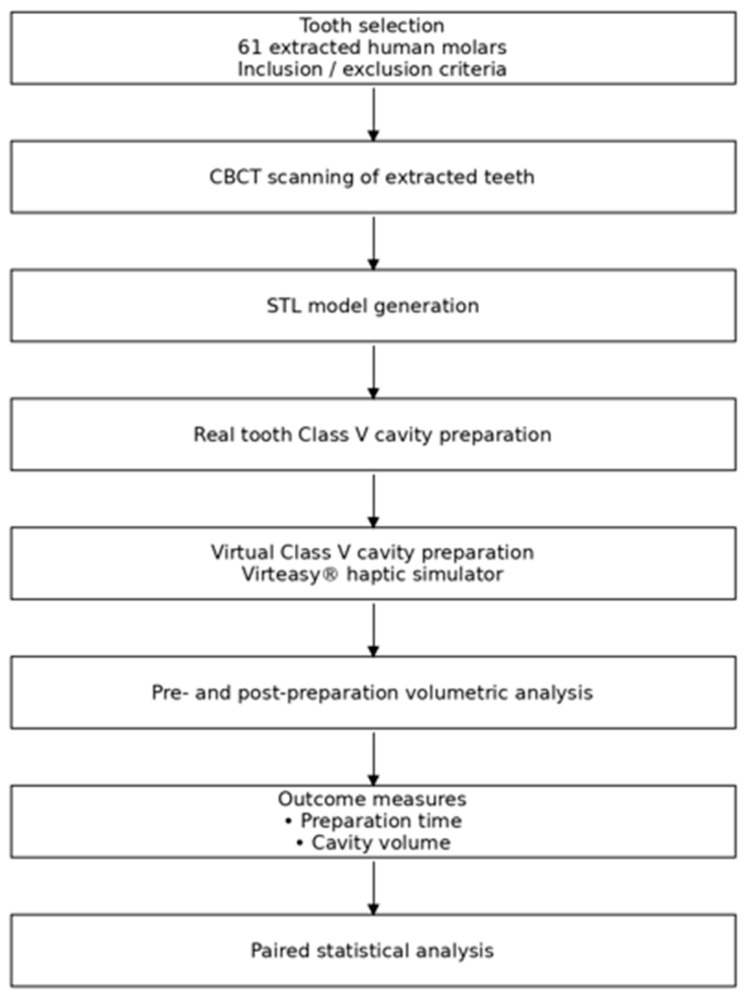
Experimental workflow of the study.

The experimental workflow is summarized in Figure 1.

Flowchart illustrating the experimental workflow of the study, including tooth selection, CBCT digitization, STL model generation, real and virtual Class V cavity preparations, volumetric analysis, and paired statistical comparison (Figure 1).

As indicated in the schematic overview of the experimental workflow, extracted human molars were scanned using CBCT to generate pre-operative STL models. Virtual replicas were imported into the haptic simulator for standardized Class V preparation. Real preparations were performed on the natural teeth, followed by a second CBCT scan to obtain post-operative STL files. Cavity volumes were quantified by digital superimposition for real teeth and automatically by the simulator for virtual preparations. Comparative statistical analyses were performed on paired data (Figure 1).

### 2.5. Statistical Analysis

No formal a priori power calculation was performed, as the sample size was determined by the available cohort. A post hoc power analysis was therefore conducted based on the observed effect sizes to estimate the achieved statistical power for the primary paired comparisons.

Statistical analyses were performed using R (v4.2.2; R Foundation for Statistical Computing, Vienna, Austria). Normality of continuous variables was assessed using the Shapiro–Wilk test and visual inspection of Q–Q plots. As all variables approximated a normal distribution, paired Student’s t-tests were used to compare preparation time and cavity volume between real and virtual preparations, with significance set at α = 0.05. Effect sizes were calculated using Cohen’s d for paired samples.

Agreement between real and virtual cavity volumes was evaluated using Bland–Altman analysis with 95% limits of agreement, and proportional bias was assessed by linear regression of the residuals. Pearson’s correlation coefficient (r) and a two-way mixed-effects intraclass correlation coefficient (ICC, absolute agreement) were calculated to assess association and reliability between modalities.

A derived efficiency index (volume removed per minute) was compared using paired t-tests. Sensitivity analyses were conducted by repeating primary comparisons after removal of extreme values (>1.5 × interquartile range). Possible order-related learning or fatigue effects were explored using linear regression of paired differences against preparation sequence.

A post hoc power analysis was conducted to estimate the achieved statistical power for the primary paired comparisons. Based on the observed effect size for preparation time (Cohen’s d = 1.08) and a sample size of 61 paired observations, the analysis indicated an excellent statistical power exceeding 0.99. In contrast, the observed effect size for cavity volume was smaller (Cohen’s d = 0.31), resulting in a moderate statistical power of approximately 0.66.

## 3. Results

### 3.1. Sample Characteristics

Preparation time was significantly shorter using the virtual simulator compared with real-tooth preparation (1.68 ± 0.71 vs. 0.98 ± 0.64 min; mean difference = −0.70 min, 95% CI: −0.88 to −0.52; *p* < 0.001), representing a large effect size (Cohen’s d = 1.08). Sensitivity analysis excluding the two highest virtual-time values yielded consistent results (*p* < 0.001; Cohen’s d = 1.02).

**Figure 2:**
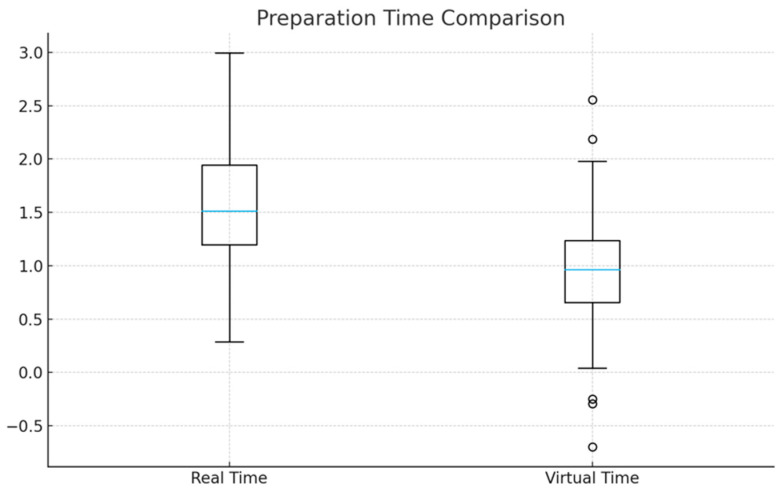
Comparison of preparation time between real and virtual cavity preparations.

Boxplots were constructed to show the distribution of preparation times (minutes) for conventional real-tooth preparations and virtual preparations performed on a haptic simulator (*n* = 61 pairs). Boxes represent interquartile range (IQR), horizontal lines indicate medians, and whiskers show 1.5 × IQR. Virtual preparation required significantly less time than real-tooth preparation (*p* < 0.001) (Figure 2).

#### 3.1.1. Cavity Volume

Virtual preparations resulted in slightly larger cavity volumes than real preparations (7.20 ± 1.48 vs. 6.44 ± 1.54 mm^3^; mean difference = +0.76 mm^3^, 95% CI: 0.01 to 1.51; *p* = 0.047), corresponding to a small-to-moderate effect size (Cohen’s d = 0.31). Variability did not differ significantly between modalities (Levene’s test *p* = 0.29).

**Figure 3:**
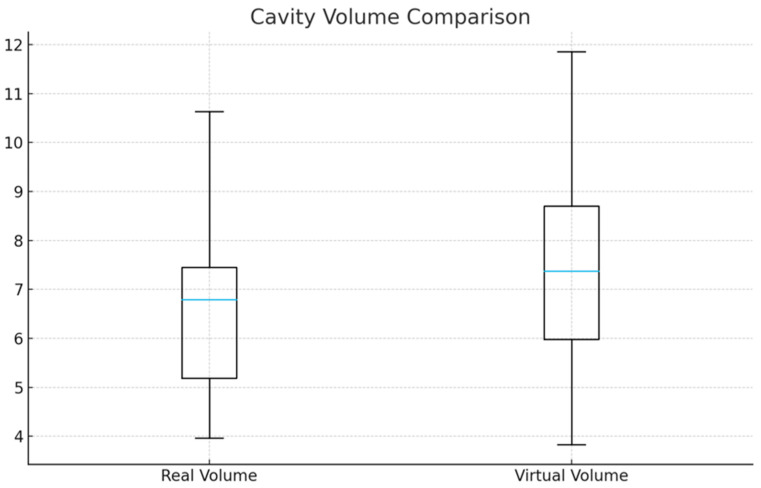
Comparison of cavity volume between real and virtual preparations.

Boxplots were constructed to show the distribution of cavity volumes (mm^3^) for conventional real-tooth preparations and virtual preparations performed using a haptic simulator (*n* = 61 paired observations). Boxes represent the interquartile range (IQR), horizontal lines indicate medians, and whiskers denote 1.5 × IQR (Figure 3).

A comprehensive summary of the primary outcomes, including descriptive statistics, mean paired differences, 95% confidence intervals, *p*-values, and effect sizes, is presented in Table 1.

#### 3.1.2. Agreement Analysis Between Real and Virtual Volumes

**Figure 4:**
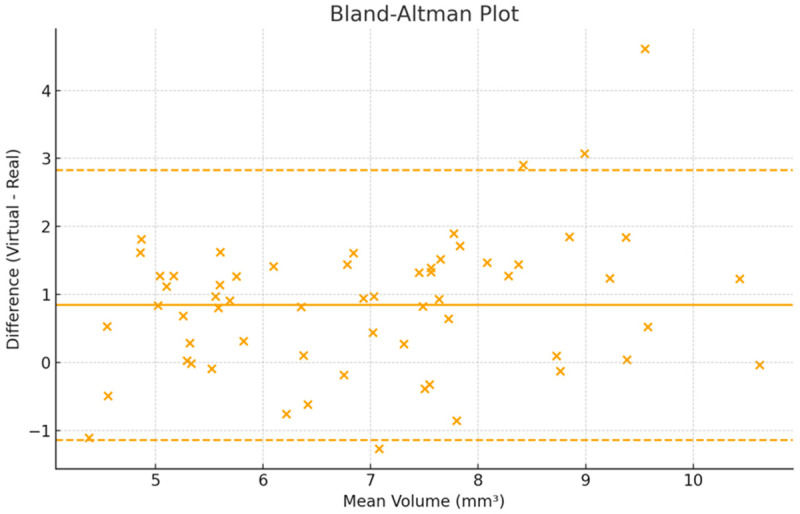
Bland–Altman plot showing agreement between real and virtual cavity volumes. The solid line represents the mean bias, and dashed lines indicate the 95% limits of agreement (±1.96 SD).

A Bland–Altman analysis showed a mean bias of +0.76 mm^3^ (virtual > real), with 95% limits of agreement ranging from −2.58 to +4.10 mm^3^. Linear regression of the differences against the means revealed no proportional bias (slope *p* = 0.41) (Figure 4).

#### 3.1.3. Correlation Between Real and Virtual Performance

**Figure 5:**
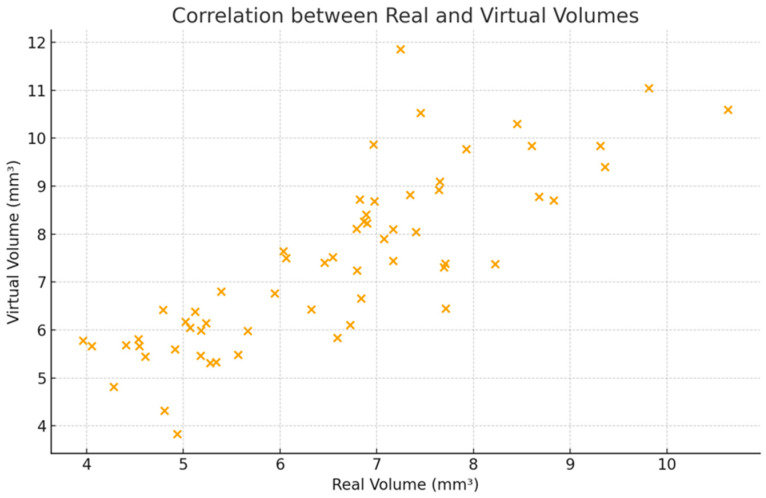
Scatter plot showing the correlation between real and virtual cavity volumes (mm^3^) for paired preparations (*n* = 61).

Pearson correlation analysis demonstrated a moderate association between real and virtual cavity volumes (r = 0.62, 95% CI: 0.43–0.76; *p* < 0.001). The intraclass correlation coefficient (two-way mixed-effects model, absolute agreement) was 0.61 (95% CI: 0.44–0.74) (Figure 5).

## 4. Discussion

The present study demonstrated that preparations performed using a haptic virtual simulator are more time-efficient and produce slightly larger cavity volumes compared to conventional preparations on extracted teeth. These results contribute to the growing body of literature supporting the integration of virtual-haptic technology in preclinical dental education. However, they also underscore the need for careful interpretation regarding clinical equivalence, skill transfer, and educational design.

### 4.1. Time Efficiency and Implications

Our finding of a significantly reduced preparation time with virtual simulation aligns with prior work showing improved operative speed after haptic training [20,21]. For example, a systematic review by Bandiaky et al. [10,11] found that haptic simulators significantly enhance psychomotor skill acquisition in preclinical dental training. Reduced time may reflect both the standardized nature of virtual tasks and the simulator’s immediate feedback features. From an educational standpoint, shorter task durations allow for increased practice frequency, which may support deliberate practice and faster competency attainment. Nonetheless, the quality of preparation must remain the focus; speed alone is insufficient if precision or tissue preservation is compromised.

While reduced preparation time may be viewed as an advantage of haptic virtual simulation, it is important to consider its potential implications for cognitive processing and procedural understanding, particularly in novice learners. In conventional preclinical laboratories, students are often encouraged to proceed at a natural pace that allows deliberate planning, continuous self-monitoring, and instructor-guided reflection. In contrast, the accelerated performance observed in virtual environments may partially reflect the absence of clinical stressors and the perception of reduced risk, which could inadvertently promote a focus on task completion speed rather than on procedural comprehension and decision-making. From an educational perspective, excessive emphasis on speed may hinder the consolidation of foundational concepts if not appropriately scaffolded. Therefore, the time efficiency associated with haptic simulation should be interpreted as a potential educational advantage only when integrated within structured curricula that emphasize accuracy, feedback, and reflective learning, rather than speed alone.

To address this concern, we provide a more balanced interpretation of the results. Specifically, the heterogeneity of the literature on haptic and virtual simulation in dental education is now explicitly acknowledged. Several studies have reported significant effects of simulation modality, level of immersion, and training sequence on skill acquisition, particularly when more complex operative or endodontic procedures are assessed and when learning outcomes are evaluated using longitudinal study designs. For example, the integration of haptic simulation with immersive virtual reality environments has been shown to improve accuracy, internal geometry, and skill transfer in endodontic access cavity preparation compared with less immersive modalities [10,11]. In addition, systematic reviews and controlled studies have highlighted that the sequence of training—such as simulation-first versus conventional-first approaches—can significantly influence performance outcomes and the consolidation of psychomotor skills [22,23]. These findings contrast with studies reporting minimal or no differences in short-term or low-complexity tasks, underscoring that the educational impact of simulation is highly context-dependent and influenced by task complexity, learner experience, and study design.

In addition, the absence of statistically significant differences in some outcomes must be interpreted in light of the study’s statistical constraints. Given that the sample size was determined by cohort availability rather than by an a priori power calculation, the possibility of Type II error cannot be fully excluded, particularly for outcomes associated with small effect sizes. Accordingly, we have intentionally avoided overinterpreting non-significant findings and refrained from framing them as evidence of equivalence between modalities. Instead, these results should be viewed as inconclusive within the limits of the present design and as a basis for generating hypotheses to be tested in larger, adequately powered studies.

Importantly, the conclusions drawn from the present study should not be interpreted as directly applicable to the acquisition of complex endodontic competencies. The experimental task evaluated—a standardized Class V cavity preparation performed over a short time frame—represents a relatively low-complexity operative procedure. Advanced endodontic skills such as canal negotiation, shaping, and obturation involve additional spatial, tactile, and cognitive demands, as well as error-sensitive decision-making processes, which typically require repeated, longitudinal practice and structured training sequences. Therefore, while the present findings support the flexible integration of haptic simulation within preclinical operative dentistry, caution is warranted against extrapolating these results to more advanced endodontic training contexts without dedicated longitudinal evidence.

### 4.2. Volume Differences and Skill Transfer

The slightly larger cavity volumes observed with virtual preparations warrant nuanced interpretation. The positive bias in the Bland–Altman analysis suggests that users may remove more tissue in the virtual environment, possibly due to lower perceived risk or differential force feedback calibration. Literature on skill transfer in VR-haptic contexts suggests that over-preparation or non-clinical cutting patterns may emerge in simulation unless strict fidelity and realism are maintained [24]. For instance, a qualitative study by Daud et al. [2,9] reported that students perceive the realism of VR-haptic simulators as still lacking, particularly in tactile feedback, which may influence performance behavior. Hence, while the volume difference is statistically significant, its clinical relevance appears small (Cohen’s d ≈ 0.31), and it should be framed as a potential risk rather than a definitive drawback.

### 4.3. Correlation, Reliability and Educational Validity

The moderate Pearson correlation (r ≈ 0.62) and moderate ICC (~0.61) between real and virtual volumes indicate partial transferability of performance patterns across modalities. This is consistent with the idea that haptic simulation provides a learning scaffold, but not a full replacement for real-tooth experience [22]. From a pedagogical viewpoint, this suggests that simulation can accelerate basic skill acquisition, but subsequent translation to natural-tooth work still requires supervision, real-world feedback, and refinement. Given the evidence from Koolivand et al. [22] indicating that VR-based education yields improved motor skills but still needs robust assessment of transfer to real settings, our data further support a blended-training model.

### 4.4. Efficiency Index and Curriculum Design

The derived efficiency index (volume removed per minute) showing superior performance in the virtual environment suggests that the simulator environment may shift behavior toward high-throughput rather than clinically conservative preparation. Educators should consider embedding specific fidelity checks and feedback mechanisms in simulation curricula to align virtual behavior with optimal clinical standards, rather than purely speed-based metrics. This resonates with the review by Felszeghy et al. [23], which emphasized that while VR-haptics increases engagement and motivation, validation and careful curriculum integration remain critical for effectiveness.

### 4.5. Limitations and Future Research Directions

Several limitations of the present study should be acknowledged. First, the experimental task was limited to a single, standardized Class V cavity preparation, which represents a relatively low-complexity operative procedure. While this approach allowed for controlled comparisons and reduced procedural variability, it does not capture the additional spatial, tactile, and cognitive demands associated with more complex operative tasks, such as Class II cavity preparations. As such, the findings cannot be directly extrapolated to more complex or multistep dental procedures. Future investigations incorporating higher-complexity procedures will be essential to more comprehensively assess the robustness and educational value of haptic virtual simulation systems. In addition, the study was conducted at a single institution and involved a single experienced instructor, which may limit the generalizability of the findings despite ensuring procedural standardization. Second, the study design was short-term and did not assess learning progression or skill retention over time. Third, the investigation was conducted in a preclinical setting, which does not fully capture the cognitive, ergonomic, and stress-related factors present in real clinical environments. Although a post hoc power analysis indicated adequate power for detecting differences in preparation time, the study may have been underpowered to detect small volumetric differences. Additionally, haptic feedback parameters were set according to the manufacturer’s default recommendations. All virtual preparations were completed in single uninterrupted sessions using a non-immersive simulator configuration; therefore, the potential effects of multiple sittings, immersive head-mounted displays, and operator fatigue were not specifically assessed and warrant further investigation. Finally, the present study did not include a cost–value or cost-effectiveness analysis, which is an important consideration for the large-scale adoption and global implementation of haptic simulation technologies and should be addressed in future research.

### 4.6. Educational and Clinical Implications

From an educational standpoint, our results support the use of haptic virtual simulation as a valid adjunctive tool in preclinical operative dentistry. Simulation can reduce faculty supervision time, offer standardized and reproducible tasks, and allow repeated safe practice—aligning with broader trends in competency-based education [23,24]. However, we caution against viewing it as a replacement for real-tooth preparation at this stage. The slight volumetric bias and moderate reliability point to the continued importance of integrated curricula, where simulation precedes real-tooth work, enabling students to hone speed and gross technique virtually, followed by refinement and verification on real substrates.

The educational value of VR-based simulators is consistent with previous research demonstrating that stereoscopic depth cues and realistic hand–tool alignment significantly improve skill acquisition and transfer in dental simulation environments [25,26].

Clinically, faster preparation times may translate into more productive preclinical labs, enabling students to engage in higher-fidelity tasks sooner. Yet, educators must ensure that time gains do not compromise tissue conservation, internal geometry or anatomical fidelity, which underpin long-term clinical competence.

### 4.7. Clinical Relevance

The findings of this study indicate that haptic virtual simulation provides a time-efficient and standardized environment for developing operative skills while producing cavity volumes that are largely comparable to those obtained on natural teeth. These results support the use of virtual simulation as a safe and effective adjunct in early preclinical training, allowing students to refine basic psychomotor abilities before transitioning to real-tooth preparations. The slight volumetric bias observed highlights the importance of blended curricula, where virtual practice enhances foundational skills without replacing real substrate experience.

## 5. Conclusions

In summary, this study shows that haptic virtual simulation provides significant time-efficiency advantages and produces cavity volumes broadly comparable to those obtained through conventional preparation, albeit with a slight bias toward greater volume removal. The moderate correlation and reliability observed between modalities support the role of simulation in early skill acquisition while also reinforcing the necessity of subsequent transition phases involving real-tooth practice. As dental education increasingly incorporates digital technologies, these findings highlight both the potential benefits and the important limitations of integrating haptic simulation into preclinical operative dentistry curricula.

## Figures and Tables

**Table 1:** Summary of Main Outcomes.

Outcome	Real Preparation (Mean ± SD)	Virtual Preparation (Mean ± SD)	Mean Difference (V−R)	95% CI	*p*-Value	Cohen’s d	Interpretation
Preparation Time (min)	1.68 ± 0.71	0.98 ± 0.64	−0.70	−0.88 to −0.52	<0.001	1.08	Large effect
Cavity Volume (mm^3^)	6.44 ± 1.54	7.20 ± 1.48	+0.76	0.01 to 1.51	0.047	0.31	Small–moderate
Efficiency Index (mm^3^/min)	3.83 ± 1.12	7.35 ± 3.41	+3.52	2.67 to 4.38	<0.001	1.21	Large effect
Correlation (r)	—	—	—	0.43 to 0.76	<0.001	—	Moderate
ICC (absolute agreement)	—	—	—	0.44 to 0.74	—	—	Moderate reliability

CI Confidence interval.

## Data Availability

The data supporting the findings of this study are not publicly available due to ethical and privacy restrictions. The dataset is securely stored at ADEMA–Escuela Universitaria under the supervision of the Data Protection Officer, Ángel Arturo López González. The data presented in this study are available on request from the corresponding author due to ethical, legal, and institutional limitations related to the handling of human tissues and medical imagingdata.

## References

[1] Farag A., Hashem D. (2021). Impact of the Haptic Virtual Reality Simulator on Dental Students’ Psychomotor Skills in Preclinical Operative Dentistry. Clin. Pract..

[2] Daud A., Matoug-Elwerfelli M., Daas H., Zahra D., Ali K. (2023). Enhancing learning experiences in pre-clinical restorative dentistry: The impact of virtual reality haptic simulators. BMC Med. Educ..

[3] Stoilov L., Stephan F., Stark H., Enkling N., Kraus D., Stoilov M. (2024). Efficacy of Virtual Preparation Simulators Compared to Traditional Preparations on Phantom Heads. Dent. J..

[4] Al-Saud L.M. (2021). The utility of haptic simulation in early restorative dental training: A scoping review. J. Dent. Educ..

[5] Rodrigues P., Nicolau F., Norte M., Zorzal E., Botelho J., Machado V., Proença L., Alves R., Zagalo C., Lopes D.S. (2023). Preclinical dental students self-assessment of an improved operative dentistry virtual reality simulator with haptic feedback. Sci. Rep..

[6] Dzyuba N., Jandu J., Yates J., Kushnerev E. (2025). Virtual and augmented reality in dental education: The good, the bad and the better. Eur. J. Dent. Educ..

[7] Gramatges-Rojas A., Sittoni-Pino M.F., Flacco N., Musalem-Dominguez O., Spinelli M., Felszeghy S., Arias-Herrera S. (2025). Can haptic reinforced VR simulation transform preclinical pulpotomy training? Insights into skill acquisition, student perceptions, and educational impact: Randomized controlled trial. Front. Oral Health.

[8] Ren J.G., Li R.F., Zhang W., Yu Z.L., Chen G. (2025). Effect of digital virtual reality simulator on pre-clinical dental surgical skill training: A retrospective study. BMC Med. Educ..

[9] Daud A., Matoug-Elwerfelli M., Khalid A., Ali K. (2024). The impact of virtual reality haptic simulators in pre-clinical restorative dentistry: A qualitative enquiry into dental students’ perceptions. BMC Oral Health.

[10] Bandiaky O.N., Lopez S., Hamon L., Clouet R., Soueidan A., Le Guehennec L. (2024). Impact of haptic simulators in preclinical dental education: A systematic review. J. Dent. Educ..

[11] Bandiaky O.N., Loison V., Volteau C., Crétin-Pirolli R., George S., Soueidan A., Le Guehennec L. (2025). Benefits of using immersive virtual reality in haptic dental simulation for endodontic access cavity training: A comparative crossover study. Int. Endod J..

[12] Shetty S., Errichetti A., Narasimhan S., Al-Daghestani H., Shetty G. (2025). The use of virtual reality and haptics in the training of students in restorative dentistry procedures: A systematic review. Korean J. Med. Educ..

[13] Patil S., Bhandi S., Awan K.H., Licari F.W., Di Blasio M., Ronsivalle V., Cicciù M., Minervini G. (2023). Effectiveness of haptic feedback devices in preclinical training of dental students-a systematic review. BMC Oral Health.

[14] Dai Q., Davis R., Hong H., Gu Y. (2025). A Comparative Pilot Study of Computer-Based Evaluation Software Versus Traditional Evaluation in Preclinical Operative Procedures. J. Dent. Educ..

[15] Faul F., Erdfelder E., Lang A.G., Buchner A. (2007). G*Power 3: A flexible statistical power analysis program for the social, behavioral, and biomedical sciences. Behav. Res. Methods.

[16] Ghamri M., Dritsas K., Probst J., Jäggi M., Psomiadis S., Schulze R., Verna C., Katsaros C., Halazonetis D. (2023). Accuracy of facial skeletal surfaces segmented from CT and CBCT radiographs. Sci. Rep..

[17] Aboelnor M.M., Kamel M.A. (2025). Cavity preparation and cementation of indirect adhesive posterior restorations using three different composite resins with a one-year follow-up: A clinical report. J. Prosthet. Dent..

[18] (2004). Dentistry—Coding System for Rotary Instruments—Part 1: Identification Code.

[19] Martínez Quiñones J.V., Orduna Martínez J., Pinilla Arias D., Bernal Lecina M., Consolini Rossi F., Arregui Calvo R. (2024). Systematic review of the utility and limits of 3D printing in spine surgery. Neurocirugia.

[20] Guyatt G., Oxman A.D., Akl E.A., Kunz R., Vist G., Brozek J., Norris S., Falck-Ytter Y., Glasziou P., DeBeer H. (2011). GRADE guidelines: 1. Introduction-GRADE evidence profiles and summary of findings tables. J. Clin. Epidemiol..

[21] Ziane-Casenave S., Mauroux M., Devillard R., Kérourédan O. (2022). Influence of practical and clinical experience on dexterity performance measured using haptic virtual reality simulator. Eur. J. Dent. Educ..

[22] Koolivand H., Shooreshi M.M., Safari-Faramani R., Borji M., Mansoory M.S., Moradpoor H., Bahrami M., Azizi S.M. (2024). Comparison of the effectiveness of virtual reality-based education and conventional teaching methods in dental education: A systematic review. BMC Med. Educ..

[23] Felszeghy S., Mutluay M., Liukkonen M., Flacco N., Bakr M.M., Rampf S., Schick S.-G., Mushtaq F., Sittoni-Pino M.F., Ackerman K. (2025). Benefits and challenges of the integration of haptics-enhanced virtual reality training within dental curricula. J. Dent. Educ..

[24] Rodrigues P., Esteves A., Botelho J., Machado V., Zagalo C., Zorzal E.R., Mendes J.J., Lopes D.S. (2022). Usability, acceptance, and educational usefulness study of a new haptic operative dentistry virtual reality simulator. Comput. Methods Programs Biomed..

[25] Kaluschke M., Yin M.S., Haddawy P., Suebnukarn S., Zachmann G. (2023). The effect of 3D stereopsis and hand-tool alignment on learning effectiveness and skill transfer of a VR-based simulator for dental training. PLoS ONE.

[26] Riutord-Sbert P., Tomás-Gil P., Pereira T.C., Szupiany-Janeczek T., Barkvoll P., López-González Á.A., González-Carrasco D. (2023). Aplicación del metaverso como técnica de aprendizaje en el grado de odontología. Estudio preliminar. Acad. J. Health Sci..

